# Bacterial bioluminescence for the real time and *in situ* monitoring of endoscope disinfection using plasma activated water

**DOI:** 10.1128/aac.01380-25

**Published:** 2026-01-30

**Authors:** Naomi Northage, Joshua A. C. Steven, Darren M. Reynolds, Malcolm J. Horsburgh, James L. Walsh, Robin M. S. Thorn

**Affiliations:** 1 Department for Gaseous Electronics, Jožef Stefan Institute 61790, Ljubljana, Slovenia; 2 School of Applied Sciences, College of Health, Science and Society, University of the West of England 1981, Bristol, United Kingdom; 3 Infection Biology & Microbiomes, Institute of Infection, Veterinary and Ecological Sciences, University of Liverpool, Liverpool, United Kingdom; 4 York Plasma Institute, School of Physics, Engineering & Technology, University of York 8748, York, United Kingdom; University of Pennsylvania Perelman School of Medicine, Philadelphia, Pennsylvania, USA

**Keywords:** cold atmospheric plasma, endoscope disinfection, plasma activated water, bioluminescence, biofilm

## Abstract

Endoscope reprocessing is a time-consuming, multi-step process, and ongoing microbial surveillance is necessary to ensure effective reprocessing and safe storage. Despite this, traditional surveillance methods, such as cultures, are not often carried out due to time and cost constraints, providing only delayed end point results from specific locations, with no insight into biofilm formation, disinfection efficacy, or any variability related to device design. Within this study, the efficacy of plasma activated water (PAW) disinfection (and subsequent regrowth) within endoscopic test pieces was investigated using bacterial bioluminescence enabling real time and *in situ* monitoring of biofilm formation and treatment efficacy. Real time imaging of bioluminescent *Pseudomonas aeruginosa* PAO1 SEI MCS5-lite was used to track biofilm growth within translucent PVC endoscopic test pieces across regions of interest using analysis of bioluminescent intensity. Results demonstrated that biofilm accumulation was more prominent around connectors compared to other regions of the test pieces. Disinfection with PAW achieved a significant 96.45% reduction in biofilm density (determined by culture) and a 93.08% reduction in bioluminescence (relative light units). However, percentage reduction in bioluminescence ranged from 77% to 95% across regions indicating lack of uniformity of treatment. Subsequent testing with plasma activated disinfectant (PAD) showed consistent biofilm reduction across all regions, with no variability between connectors and other regions as observed with PAW and other treatments. In conclusion, bioluminescence can be used to assess efficacy of disinfectants and effect of endoscope design by tracking biofilm density in real time and *in situ*.

## INTRODUCTION

Endoscope reprocessing is a time-consuming, multi-step process consisting of manual cleaning, high-level disinfection (HLD), rinsing, drying, and finally storage (1). Endoscope reprocessing has improved over the past decade with widespread use of automated endoscope reprocessors (AERs) for HLD; however, HLD of flexible endoscopes has recently come under scrutiny due to the continued evidence of microbial contamination found in samples taken from “patient-ready” reprocessed flexible endoscopes (2, 3). Other factors such as instrument damage, incomplete drying, and improper storage all can contribute to increased microbial contamination and biofilm formation within the inner channel system resulting in failures in HLD (3–5). Studies have shown that surface roughness of used flexible endoscope materials increases over time and that bacterial attachment increases with roughness, ultimately decreasing the efficacy of HLD (6, 7). Immediately after HLD, it is suggested that endoscopes are flushed with alcohol and air-dried for 10 min before hanging and storing in a drying cabinet (8, 9). The drying procedures of flexible endoscopes can vary both across countries and even within countries, whereby reprocessing guidelines often do not include the drying step, and studies have shown low adherence to manual drying steps, meaning endoscopes may be stored wet (4, 10). Proper storage is necessary to ensure microbial regrowth does not occur within the recommended time period of storage before use (4, 11). There is disagreement regarding the maximum time period an endoscope can be stored before it is assumed there will be unsafe levels of microbial contamination present (12, 13). A systematic review by Schmelzer et al. found that the safe storage time of flexible endoscopes ranged from 2 to 56 days, and concluded endoscopes could be stored for 7 days, but that ongoing surveillance was necessary (13).

There are several methods of microbiological endoscope surveillance, such as surveillance cultures, ATP bioluminescence, and PCR-based detection (14). The gold standard for surveillance is cultures which involves swabbing channels or flushing with sterile saline fluid before culturing; however, it is rarely performed routinely due to time constraints (15). ATP bioluminescence testing is currently used as a method of surveillance in flexible endoscopes, as this provides faster results, whereby the sample surface is swabbed, with the swab then placed in a reaction tube and the relative light units (RLU) read immediately using a luminometer (16). However, it only gives insight into specific sampling sites and lacks specificity for differentiating viable microorganisms from organic material. PCR-based methods detect microbial DNA/RNA with high sensitivity but may also detect non-viable organisms (17).

Bacterial bioluminescence is a dynamic approach that could be used for modeling the efficacy of endoscope reprocessing, enabling the monitoring of biofilm formation, efficacy of novel disinfection methods, and subsequent regrowth, both *in situ* and in real time. Bacterial bioluminescence has been applied to various microbiological phenomena, including monitoring of bacterial growth, effects of biocides, and pathogenesis (18–20). Predominantly, bioluminescence is applied to the study of bacterial infection models (21). For example, cloning of the bioluminescence (lux) operon into infectious pathogens and visualizing in real time the progression of the infection in the gastrointestinal tract of mice *in situ* (21). In this process, bacteria are engineered to express the *lux* operon consisting of the *luxCDABE* gene cassette, obtained from *Photorhabdus* species, enabling light emission (18, 19, 22–26). The production of light is used as a reporter of viable, metabolically active cells, as the biochemical pathways involved in bioluminescence are dependent on the production of ATP and NADPH from cellular metabolism (27). In the context of endoscope HLD, bioluminescence can be used as a real time monitor of biofilm formation, the efficacy of novel endoscope disinfection methods, and subsequent regrowth. In addition, contamination can be tracked in regions of interest before, during, and after disinfection.

A novel endoscope disinfection method that could benefit from real time monitoring is plasma activated water (PAW). PAW is produced by exposing water to a plethora of reactive oxygen and nitrogen species (RONS) generated from cold atmospheric pressure plasma (CAP). Preliminary studies have highlighted the potential of CAP and PAW as methods of endoscope disinfection (28, 29). However, it has been highlighted that CAP may not fully reach or penetrate throughout the endoscopic channel particularly when soil aggregates are present (29). Therefore, PAW and activation of other liquids are being explored. The efficacy of PAW as an antimicrobial agent has been widely detailed, and some studies have shown it to be capable of significant reduction in biofilms, suggesting it as a promising alternative to traditional disinfection methods (30). Notably, Suwal et al. have described the use of bioluminescence for characterization of CAP and PAW bacterial inactivation in food processing applications (31). In this study, the efficacy of plasma activated water (PAW) disinfection (and subsequent regrowth) within endoscopic test pieces was investigated using bacterial bioluminescence enabling real time and *in situ* monitoring of biofilm formation, treatment efficacy, and any subsequent microbial regrowth.

## MATERIALS AND METHODS

### Bioluminescent reporter strain and growth conditions

A bioluminescent reporter strain, *P. aeruginosa* PAO1 SEI MCS5-lite, produced by transformation of *P. aeruginosa* PAO1 SEI (ATCC 15692) with a recombinant plasmid containing the *luxCDABE* gene cassette of *Photorhabdus luminescens,* was used in this study (18, 32). *P. aeruginosa* was chosen as an example of a model biofilm-forming pathogen, known for persistence in clinical settings (33). Figure S1 depicts the genes encoding bioluminescence within the *lux* operon integrated into the recombinant plasmid. *P. aeruginosa* PAO1 SEI MCS5-lite, herein referred to as PAO1 plite, was cultured and maintained on tryptic soy agar (TSA) containing 10 mg/L gentamicin (Sigma-Aldrich, Dorset, United Kingdom), the selective agent for the recombinant plasmid. The bioluminescent activity of PAO1 plite was checked prior to any experimental runs (Fig. S2). A single colony of PAO1 plite was used to inoculate 10 mL of tryptic soy broth (TSB) with 10 mg/L gentamicin and left to incubate overnight, and the concentration was then adjusted by broth dilution to 1 × 10^6^ CFU/mL.

### Biofilm formation within endoscopic test pieces

**Fig 1:**
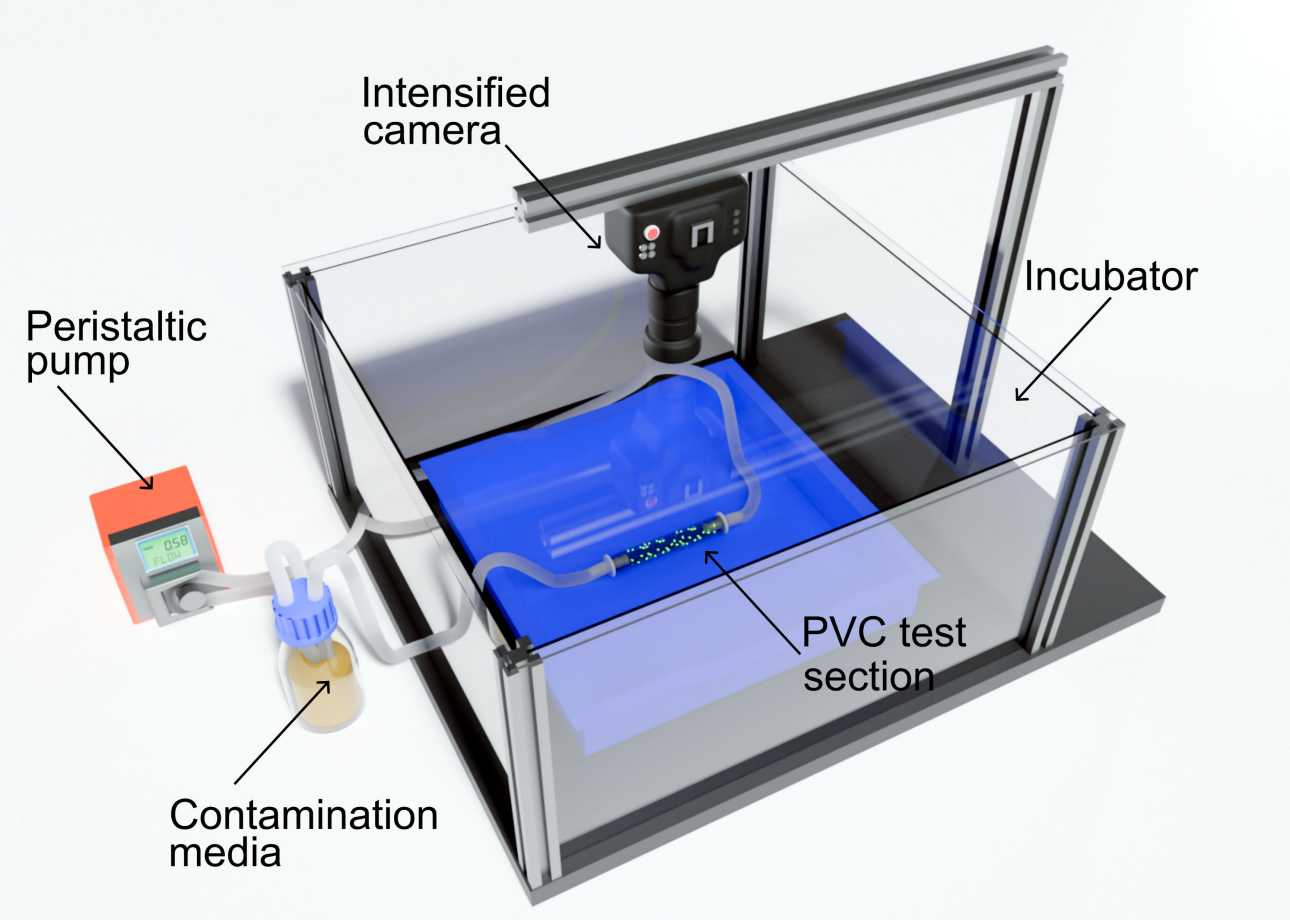
Diagram of flow system used to contaminate translucent PVC endoscopic test pieces with bioluminescent *P. aeruginosa* PAO1 SEI MCS5-lite (PAO1 plite) using a peristaltic pump operating at 100 mL/min. The system was set up within an incubator with an EMCCD camera mounted in view of the endoscopic test pieces to capture light output.

Endoscope surrogate test pieces were produced from 6 mm diameter translucent PVC tubing, each 10 cm in length. While Teflon is usually the material of choice, its opacity makes accurate visualization difficult; therefore, translucent PVC endoscopic test pieces were used as a substitute (34). To contaminate the endoscopic test pieces, a flow system was developed using a peristaltic pump operating at 100 mL/min allowing continuous flow of fluid through the system (Fig. 1). The flow system was set up within a blacked-out incubator set at 37°C, with a low light photon counting camera (iXon EM+ DU-897 back-illuminated EMCCD camera with a Tamron SP AF 17-35 mm F/2.8-4 lens; Andor, Belfast, UK) mounted onto the incubator and positioned directly above the test pieces for imaging. A well-established method of producing biofilm contamination representative of those found in the inner channels of flexible endoscopes was adopted, with minor modifications to contaminate the endoscopic test pieces with a 24 h PAO1 plite biofilm (35, 36). First, TSB containing 1% human serum was circulated throughout the endoscopic tests pieces at a flow rate of 100 mL/min until all test pieces were filled. Once filled, the pump was stopped and the system left to incubate at 37°C for 24 h, allowing attachment of organic matter to the inner surface of the endoscopic test pieces increasing bacterial adherence potential. To maintain selective pressure for the recombinant plasmid, TSB containing 10 mg/L gentamicin was used throughout. After the 24 h incubation, the media was drained, the system rinsed with sterile water, and fresh TSB with PAO1 plite was circulated, and finally, the contamination media was left within the flow system for 24 h at 37°C to cultivate biofilm. During biofilm formation, low light imaging was undertaken every 10 min with an exposure time of 5 min per frame for the full 24 h duration.

### Preparation and characterization of PAW

A low temperature, surface barrier discharge (SBD) plasma device was used to produce the PAW, as described in previous work (37). On application of a sufficiently high voltage to the SBD electrodes, a thin layer of air plasma formed within the hexagonal gaps of a grounded mesh stainless steel electrode. The electrode unit was positioned 5 cm above 200 mL of deionized water (Purite Analyst 40, Seuz Water) with continuous stirring to allow uniform diffusion of the long-lived reactive species generated from the plasma into the water over the 25 min activation period. A constant input power of 30 W was used; from previous work, it is known that such conditions favor the generation of reactive nitrogen species (RNS) (38). The PAW was stored at 4°C for a period of 4 days before it was used due to logistical constraints. Fourier Transform Infrared (FTIR) spectroscopy was used for characterization of the longer-lived reactive species present in the plasma effluent that would reach the liquid interface (FT/IR-4200 spectrometer, JASCO, Tokyo, Japan). Data were acquired over 25 scans with a resolution of 2.0 cm^−1^. The characteristics of the PAW solution, including the pH and concentrations of hydrogen peroxide (H_2_O_2_), nitrates (NO_3_^−^), and nitrites (NO_2_^−^), were measured over a 4-day period. The pH of the PAW solution was measured using a pH probe (Hanna Instruments 9813-6 with pH probe HI-1285-6). The H_2_O_2_ concentration within the PAW was determined according to spectrophotometry at 540 nm (SPECTROstar Nano, BMG LABTECH) in the presence of ferrous-xylenol orange, as described in the protocol by Dringen et al. (39). The concentration of NO_2_^−^ was measured based on the interaction with Griess reagent (Supelco Ltd, MFCD01866819) at 548 nm. A colorimetric assay was used to measure the concentration of NO_3_^−^ based on the interaction of nitrate ions with sodium salicylate (Sigma-Aldrich Ltd, CAS 54-21-7) in a sulfuric acid medium after evaporation and quantified using spectrophotometry at 420 nm.

### Bioluminescent monitoring of PAW disinfection and subsequent bacterial regrowth

Following contamination of the PVC endoscopic test pieces with a 24 h PAO1 plite biofilm, PAW was introduced into the inner channels to disinfect the test pieces. To achieve this, the flow system was first drained of the contamination media and rinsed with deionized (DI) water, followed immediately by continuous circulation of the PAW at 100 mL/min for a 5 min disinfection period. During the disinfection period, the system was imaged every 30 s by low light photometry using the EMCCD camera. At the end of the disinfection period, the system was once again rinsed with DI water, and the PVC endoscopic test pieces were subsequently left in position to enable bacterial regrowth to be monitored over a 24 h time period, with low light imaging undertaken every 10 min.

### Further testing of biofilm removal using bioluminescent monitoring

Further testing of biofilm removal using bioluminescent monitoring was conducted by comparing the effects of DI water, PAW, and plasma activated disinfectant (PAD). The PAD was created by plasma activating a pH-buffered peracetic acid (Olympus EndoAct/EndoDis, Olympus Surgical Technologies) prepared in a 1:1 ratio at a 2% concentration under identical plasma conditions as those described previously. Each liquid was circulated through the contaminated endoscopic test pieces in the flow system at 100 mL/min for 5 min at room temperature, whereby low light images were taken before and after the 5 min disinfection period.

### Data analysis

All experiments were conducted with at least three biological repeats and/or three technical repeats. Results are presented as mean ± standard deviation. Statistical analysis was performed using GraphPad Prism 10 and established using one-way analysis of variance (ANOVA) test. A *P*-value of ˂0.05 was considered significant. The images obtained of the bioluminescent biofilm within the PVC endoscopic test pieces were analyzed using ImageJ (40). Images were converted to 8-bit RGB mode, and channels were split to obtain the red channel of the image as presented in this work. A Gaussian Blur was applied to reduce noise and preserve features. Bioluminescence (RLU) was measured by taking the mean pixel intensities from the test pieces image or the region of interest. Background intensities were subtracted where appropriate.

## RESULTS

### Monitoring biofilm formation within endoscopic test pieces using bioluminescence

**Fig 2:**
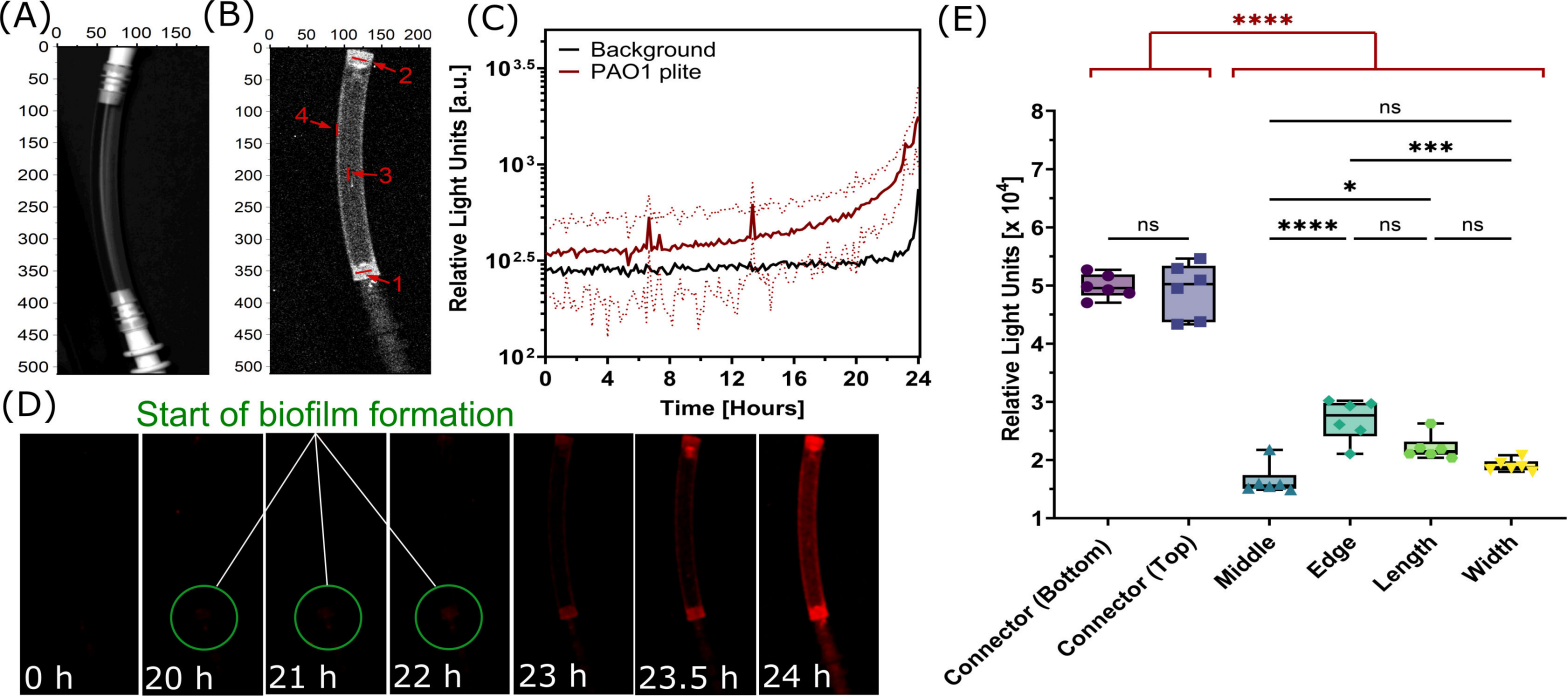
Bioluminescent biofilm formation within endoscope test pieces. (**A**) Light image of the PVC endoscopic test piece used in the experiments. (**B**) Image highlighting the regions of interest where bioluminescent signal from biofilm was measured, region 1—Connector Bottom, region 2—Connector Top, region 3—Middle, region 4—Edge. (**C**) Light output emitted by the PAO1 plite biofilm over 24 h. Dotted lines indicate the standard deviation. (**D**) Sequential bioluminescence images captured during biofilm formation from 0 to 24 h. (**E**) Comparison of light intensity across regions of interest and across the length and width of the test pieces. Data compiled from final low light images taken at 24 h. Statistical analysis: one-way ANOVA, post-hoc Tukey test, ns = not significant, *P < 0.05, ***P < 0.001, ****P < 0.0001.

In this work, investigation of biofilm formation was carried out by monitoring bioluminescent PAO1 plite contamination within PVC endoscopic test pieces (Fig. 2A) by imaging every 10 min for 24 h. Specific regions of interest were also tracked throughout biofilm formation to identify any locations more prone to biofilm formation (regions illustrated in Fig. 2B). RLU from across the length (region 5) and width (region 6) of the endoscopic test pieces were also measured. As shown in Fig. 2C, initial growth was slow until around 20 h where there was a significant increase in bioluminescence (RLU), and intensity continued to increase until the end of the 24 h biofilm formation period. This was also confirmed by the images taken of biofilm formation highlighting the start of biofilm growth at 20 h and a rapid increase in light intensity between 23 and 24 h (Fig. 2D). The images revealed that biofilm accumulation was particularly prominent around the connectors. Further analysis of the bioluminescent signal across the regions of interest of the test pieces confirmed significantly higher RLU at the connectors compared to other areas, such as the middle or edges (Fig. 2E). There was found to be a threefold difference in bioluminescence between the connectors and the middle of the test pieces, with an average of 4.95 × 10^4^ RLU for the connectors and 1.65 × 10^4^ RLU for the middle. The one-way ANOVA verified the significant differences in biofilm formation among the regions of interest (*F* = 159.7, *P* < 0.0001), with post-hoc Tukey’s test showing the highest biofilm formation in both connectors and the lowest in the middle of the endoscopic test pieces.

### PAW characteristics

PAW was used to investigate the use of bioluminescence as a tool for monitoring novel disinfection methods. The PAW generated in this investigation was dominated by RNS, which is to be expected from the type of air plasma device used. Effluent from the headspace between the plasma and liquid surface was analyzed using FTIR, and peaks were observed for N_2_O at around 2,237 cm^−1^, NO_2_ at around 1,630 cm^−1^, and N_2_O_5_ and HNO_3_ at around 1,297 cm^−1^ (Fig. S3). There was an ozone peak present after 1.5 min at around 1,055 cm^−1^; however, this was not detected at the end of the 25 min PAW generation time. Following generation, the PAW was stored at 4°C for 4 days due to logistical constraints. The pH and hydrogen peroxide, nitrite, and nitrate concentration of the PAW solution were measured every day up until use (Fig. S4). The pH of the final solution remained low with a final value of 3.02 (±0.13). The final concentration of NO_2_^−^ was 30.76 (±1.68) µM and NO_3_^−^ was 0.58 (±0.03) mM. No hydrogen peroxide was present in the final solution. The presence of nitrates, nitrites, and the low pH of the solution suggested it would still be capable of antimicrobial activity despite the storage period.

### Bioluminescence as a tool for assessing efficacy of PAW disinfection

**Fig 3:**
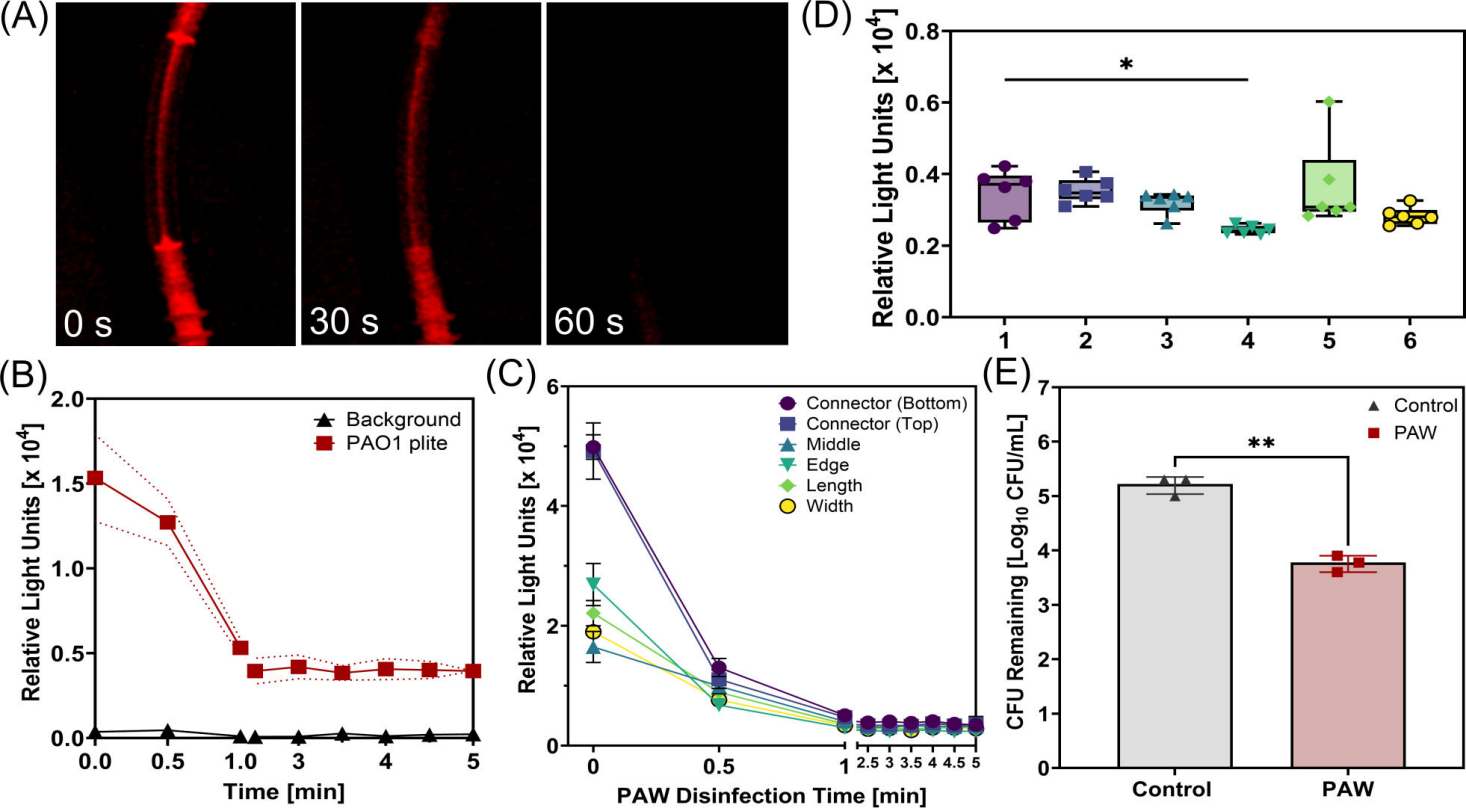
Assessing PAW disinfection against bioluminescent biofilm within endoscope test pieces. (**A**) Bioluminescence images of *P. aeruginosa* PAO1 plite biofilm at 0-, 30-, and 60-s during PAW disinfection. (**B**) Bioluminescence (relative light units) from the biofilm during the 5 min PAW treatment. Dotted lines indicate the standard deviation. (**C**) Bioluminescence (relative light units) across regions of interest during the 5 min PAW disinfection period. (**D**) Final bioluminescence (relative light units) across all regions of interest following 5 min PAW disinfection (1—Connector Bottom, 2—Connector Top, 3—Middle, 4—Edge, 5—Length, and 6—Width). (**E**) Colony-forming units remaining after 5 min of PAW treatment compared to the untreated control. Statistical analysis: one-way ANOVA, post-hoc Tukey test, *P < 0.05, **P < 0.005.

Bioluminescence was used as an *in situ* and real time tool to assess the efficacy of PAW disinfection against biofilm formation within endoscopic test pieces. The PAW was recirculated through endoscopic test pieces for a 5 min disinfection period and low light images taken at 30 s intervals, demonstrating that there was a rapid decrease in bioluminescence (RLU) within the first 60 s of PAW treatment (Fig. 3A). The RLU of the biofilm density within the endoscopic test pieces during treatment is shown in Fig. 3B, with a resultant 93.08% reduction in light output, and evidence that the PAW had the greatest effect on the biofilm within the first minute of exposure. After the first minute of exposure, RLU output from the biofilm was detected (albeit low levels) for the remainder of the disinfection step above background levels, indicating that there was still some activity within the biofilm. The RLU across regions of interest during the 5 min PAW disinfection period is shown in Fig. 3C. The initial bioluminescent values ranged from 1.51 × 10⁴ to 5.46 × 10⁴ RLU across the regions of interest and reduced significantly to final values ranging from 2.31 × 10^3^ to 6.02 × 10^3^ RLU (Fig. 3D). There was a significant difference observed between the connector (bottom) and the edge. The percentage reduction in light output ranged from 77% to 95%, with the highest reduction observed at the connector regions (~93%–95%), while the width region showed the lowest reduction (~77%), indicating spatial variability in PAW efficacy. Traditional colony counts were also conducted for comparison, and notably, there was a significant 1.45 log reduction or 96.45% reduction in PAO1 plite biofilm contamination with the 5 min PAW treatment (Fig. 3E).

### Bioluminescence for monitoring real time regrowth within endoscopic test pieces

**Fig 4:**
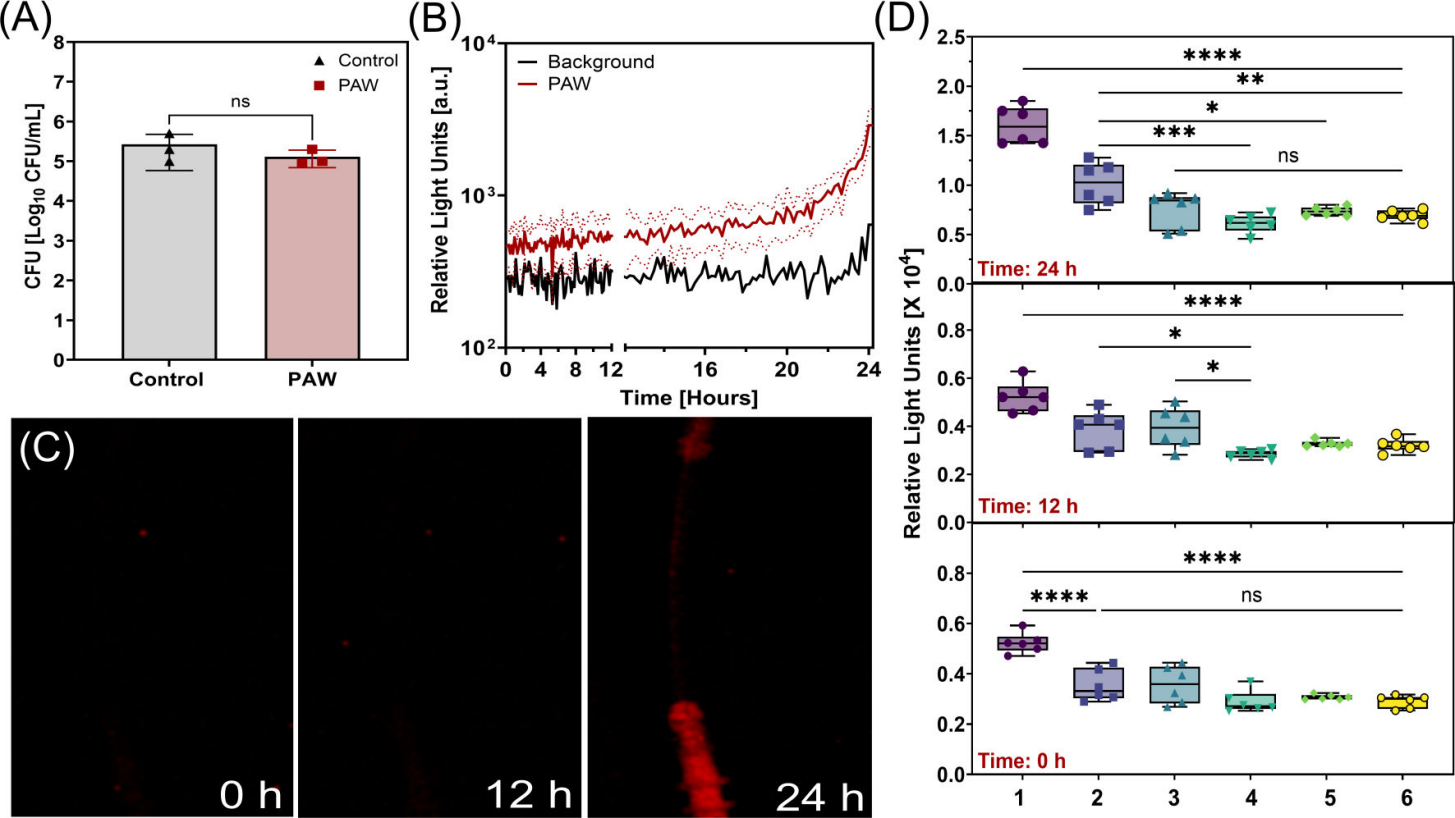
Assessing bioluminescent biofilm regrowth within endoscope test pieces following PAW treatment. (**A**) Colony-forming units of the untreated control pieces and the PAW treated test pieces after the 24-h regrowth period. (**B**) Bioluminescence monitoring of untreated control test pieces and the PAW treated test pieces over the 24-h period post-PAW treatment. Dotted lines indicate the standard deviation. (**C**) Bioluminescence images of regrowth within test pieces at 0, 12, and 24 h. (**D**) Box plots of relative light units (RLU) at 0, 12, and 24 h for specific regions of interest (1—Connector Bottom, 2—Connector Top, 3—Middle, 4—Edge, 5—Length, and 6—Width). Statistical analysis: one-way ANOVA, post-hoc Tukey test, ns = not significant, *P < 0.05, **P < 0.005, ***P < 0.001, ****P < 0.0001.

Following the PAW disinfection period, PVC test pieces were left for 24 h to monitor regrowth in real time and *in situ*. Colony counts from the test pieces showed no significant difference between the control and PAW treated samples, as the PAW treated sample returned to approximately 10^5^ CFU/mL after 24 h (Fig. 4A). There was some fluctuation in RLU of the PAW treated PVC test pieces during the regrowth step for the first 22 h, followed by a sharp increase (Fig. 4B). This was also apparent from the images taken over the regrowth period, shown in Fig. 4C. In addition, significant regrowth was observed around the connectors compared to other regions (Fig. 4D). Detailed analysis of the regions of interest over time showed that the starting measurements ranged from 2.53 × 10^3^ to 5.92 × 10^3^ RLU, being highest at the bottom connector and lowest at the width region. After 24 h, bioluminescence increased across all regions, but differences between regions were still observed with the highest increase at the connector, equating to a 3.14-fold increase to 1.85 × 10⁴ RLU, and the lowest regrowth observed across the width, equating to a 2.76-fold increase to 6.94 × 10^3^ RLU.

### Enhancing inactivation efficiency

**Fig 5:**
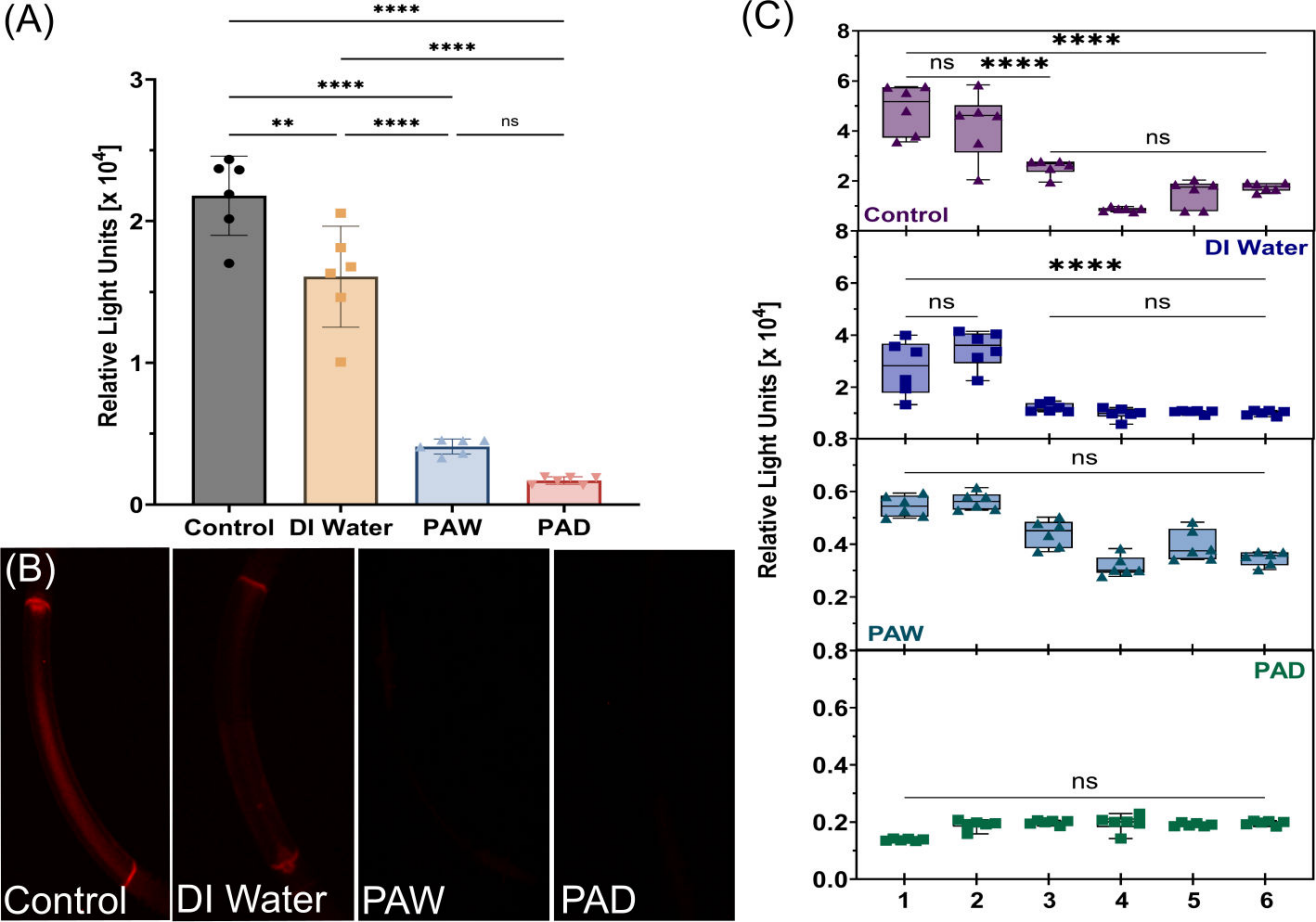
Comparative assessment of PAW and PAD against bioluminescent biofilm within endoscope test pieces.(**A**) Bioluminescence (Relative Light Units) from *P. aeruginosa* PAO1 plite contamination within untreated (control) and test pieces exposed to a 5 min DI water, PAW, or PAD treatment. (**B**) Bioluminescence images of endoscope pieces after 5 min of different treatments. (**C**) Box plots of relative light units (RLU) for specific regions of interest after each applied treatment (1—Connector Bottom, 2—Connector Top, 3—Middle, 4—Edge, 5—Length, and 6—Width). Statistical analysis: two-way ANOVA, ns = not significant, **P < 0.005, ****P < 0.0001.

Further testing of disinfection efficacy using other solutions (DI water and PAD), was carried out to compare with PAW, using the same procedure described previously. Figure 5A shows the RLU after 5 min of each treatment method. After DI treatment, the biofilm had a final light output of 1.61 × 10^4^ RLU, showing a 26.16% decrease compared to the control (2.18 × 10^4^ RLU). Both PAW and PAD treatment resulted in more dramatic decreases, with an 81.19% decrease for PAW to 4.10 × 10^3^ RLU and a 92.20% decrease for PAD to 1.70 × 10^3^ RLU. However, there was no significant difference between PAW and PAD. This can be seen from the bioluminescent images of the test pieces in Fig. 5B where both PAW and PAD treatments show minimal to no visible light, aligning with the low light output values obtained. Investigation of the regions of interest again highlighted a location-dependent level of decontamination (Fig. 5C). With a 5 min circulation of DI water, the reduction was uneven across regions with both connectors having significantly more biofilm remaining than all other regions. For PAW treatment, there was variation in light output across regions; however, it was not significant in contrast to that seen previously (see Fig. 3). PAD consistently reduced contamination across all regions of interest to comparable levels, whereby there was no differentiation between connectors and other regions as was seen for other treatments. This highlights that even though disinfectants may be capable of significant reduction in biofilm, it is necessary to ensure uniformity across all regions.

## DISCUSSION

The *lux* operon, obtained from *Photorhabdus* species, enables expression of bioluminescence within transformed strains (23, 41). The biochemical pathways involved in expression of bioluminescence are dependent on the production of ATP and NADPH from cellular metabolism; thus, light production can be used as a reporter of viable, metabolically active cells (27). Previous studies have shown that the quantifiable use of bioluminescence for cell viability shows good correlation with standard viable counts (18). The use of bacterial bioluminescence can be considered more advantageous than other measures of cell viability as it provides an insight into cellular metabolism in real time and *in situ* (20, 42). A previous study has explored the use of bacterial bioluminescence to assess the efficacy of fast acting biocides (20). And, the application of bacterial bioluminescence for characterization of CAP and PAW bacterial inactivation has been detailed by Suwal et al. using *Escherichia coli* K12 *lux,* proving insight into inactivation kinetics (31). Building upon these two important studies, this work explored the application of bacterial bioluminescence for real time and *in situ* monitoring of biofilm formation, PAW disinfection, and biofilm regrowth within narrow lumens as surrogates for endoscopic working channels.

Biofilm formation continues to be a major challenge in endoscope reprocessing, highlighting the need for new disinfection methods with antibiofilm activity. In this work, biofilm formation was undertaken within surrogate PVC endoscopic test pieces representative of the narrow lumen channels within endoscopes. It is important to note that while Teflon is the material of choice for flexible endoscopes, its opacity makes accurate visualization difficult; therefore, PVC endoscopic test pieces were used as a substitute due to their translucent nature (34). Bacterial adherence is slightly higher for PVC than Teflon; however, this can vary across species, and PVC can reliably support bacterial growth patterns comparable to those observed on clinical materials (43). Growth of the PAO1 plite biofilm was relatively slow within the developed model, whereby it appears that the bacterium was still in the log phase of growth when the final image was taken at 24 h. *P. aeruginosa* PAO1, the strain used for transformation with the bioluminescent reporter, is a well-studied model for bacterial biofilm formation due to its preference for growing as aggregates or biofilms rather than planktonic growth (44). It has been shown that maximal adherence of *P. aeruginosa* to PVC may only be reached after 24 h which could explain the slow increase seen in this investigation (45). Interestingly, there were significantly higher levels of bioluminescence around the connectors indicating higher levels of bacterial accumulation compared to other regions of interest within the test pieces (middle, edges, length, and width). The significantly higher biofilm formation around the connectors is likely due to the differences in surface roughness, flow dynamics, and shear stress (46–48). This result has also been highlighted by other studies where contamination was predominantly associated with the connecting sections (49, 50). Connectors and other endoscope reprocessing accessories must also be adequately cleaned; however, there is evidence of frequent failures to properly disinfect these components (51). In addition, difficult to reach areas may allow for any colonizing bacteria to avoid HLD. Thus, highlighting the importance of thorough investigation to identify any areas that may be more prone to biofilm accumulation, particularly when considering new endoscope designs.

The antimicrobial activity of PAW was tracked in real time and *in situ* for a contact time of 5 min. This contact time was chosen as it is the time period most often used for the disinfection stage within an automated endoscope reprocessor (52). Traditional colony counts showed a 1.45 log reduction in PAO1 plite biofilm contamination with a 5 min PAW treatment; however, an important observation is the discrepancy between the antimicrobial activity of PAW reported within this study and in previous work. In a study using the same SBD plasma device to activate water under the same conditions as detailed in this study, a 5 min PAW treatment resulted in a 3.66 log reduction of PAO1 biofilm contamination, significantly higher than that observed here (37). This decrease in antimicrobial activity is likely attributed to the 4-day storage period, which was unavoidable due to logistical constraints. Other studies have also reported that antimicrobial activity decreases over time, with Traylor et al. reporting that the pH of the solution remains low but the hydrogen peroxide and nitrite concentration diminish within a few days (53). The lower antimicrobial activity of PAW against *P. aeruginosa* PAO1 plite is most likely not due to the modification of the PAO1 strain as other studies have shown that integration of the bioluminescent reporter plasmid does not affect fitness of the strain (32). Therefore, it can be concluded that the antimicrobial activity of the PAW used in this study reduces over storage time; thus, it is recommended that where possible it is used immediately after preparation. It should also be noted that this study focused only on a single species and further work using clinically relevant mixed species biofilms must be carried out to fully evaluate efficacy.

It was observed that PAW had the greatest effect within the first minute of exposure. This is similar to most disinfectants, for example, peracetic acid, which shows the most significant microbial reduction within the first minute of exposure despite a 5 min contact time (54, 55). PAW resulted in a significant reduction in bioluminescence over the 5 min contact period. Despite significant reductions across all regions of interest, there was significant variation between the connector and other areas, ranging from 77% to 95%. This highlights that while PAW disinfection can be effective, it may not uniformly disinfect all areas. In comparison with traditional disinfectants, Bridier et al. have shown that treatment with peracetic acid causes immediate and uniform loss of fluorescence in cell clusters of *P. aeruginosa* ATCC 15442 (56). However, other disinfectants like benzalkonium chloride resulted in a nonhomogeneous loss of fluorescence within the biofilm structure (56). These results demonstrated that there are differences in the spatiotemporal patterns of biofilm inactivation. Furthermore, investigation of the action of benzalkonium chloride with clinical *P. aeruginosa* isolates Laus 3, Laus 16, and Laus 21 showed that spatial and temporal inactivation patterns differed depending on the strain (56). While peracetic acid can uniformly disinfect across a simple surface, it has been shown that achieving a uniform disinfection remains a challenge due to the complexity of endoscope designs (57). The narrow lumens, bends, and connector systems often prevent even distribution and sustained contact of disinfectants, increasing risk of residual biofilm contamination or organic debris.

Potential microbial regrowth is an important aspect of endoscope disinfection that is often overlooked. Endoscopes can only be stored for a set amount of time which can range from 2 to 56 days, but it is recommended that they are stored for no more than 7 days (13). Despite these recommendations, reprocessed endoscopes often reach unsafe levels of bacterial contamination when left overnight or over the weekend (9). Regrowth following PAW disinfection was explored and showed slight variation compared to the initial growth patterns observed during biofilm formation within the endoscope test pieces. For the initial 22 h following disinfection, a lot of fluctuation in bioluminescence was observed, followed by a sharp increase post 22 h. This could be related to bacterial cells immobilized within the core of microcolonies within the biofilm having a reduced light output due to absorption from surrounding viable and non-viable cells post-treatment (18). It is also possible that the fluctuation is indicative of the bacteria responding to the nitrosative effects of the PAW treatment and trying to recover. Further work would be required to investigate this phenomenon. However, the results of this study demonstrate that within this model system, the bacteria recover and become most metabolically active at 22 h, which is critical information when considering endoscope reprocessing procedures. In addition, final images of the bacterial growth show there is up to a 3.14-fold increase in RLU from bacterial growth over a 24 h period. Again, there was significant regrowth around the connectors, highlighting the need for ongoing surveillance from different locations within endoscopes.

Further testing was conducted to investigate any distinct patterns with different treatments, as a result of the differences observed across regions of interest with PAW and during regrowth. DI water resulted in a significant 26.16% RLU reduction compared to the control; however, the reduction was uneven across regions. The reduction in bioluminescence following DI water treatment is likely due to the mechanical force of flushing, combined with the lack of nutrients and hypotonic environment of pure water causing stress on cells (58). Both PAW and PAD showed significant decreases in light output, 81.19% and 92.20%, respectively. Notably, PAD consistently reduced biofilm density across all regions of interest to comparable levels, whereby the connectors did not have the significantly higher levels of contamination seen with other treatments. These results highlight the importance of considering both the overall antimicrobial efficacy and the uniformity of disinfection when evaluating treatment methods. While it is not intended that this method would replace traditional surveillance cultures in clinical settings, the results indicate that it would be a valuable experimental model to identify high-risk areas and support the testing of new disinfectants under controlled yet realistic conditions.

### Conclusion

In conclusion, this study highlights the potential use of bacterial bioluminescence as a tool for real time and *in situ* monitoring of biofilm formation, disinfection efficacy, and regrowth within endoscopes. Not only does the use of bacterial bioluminescence allow visual and quantifiable confirmation of significant reduction in biofilm density, but it can also be used to assess the entire process over multiple regions providing insight into effectiveness. In this study, significant biofilm formation was observed around the connectors, and while PAW had a significant 96.45% reduction in biofilm density, the reduction varied across regions from 77% to 95%. Importantly, the significant reduction seen with PAW is consistent with earlier reports, further supporting that PAW is a viable approach for HLD of endoscopes (37, 59). A sharp increase in RLU from biofilm growth was noted 22 h after PAW disinfection, and after 24 h, there were 3.14-fold higher levels of biofilm growth. This highlights the need for continuous monitoring. PAD reduced biofilm density by 92.20% and most notably reduced density across all regions of interest to comparable levels, with no significantly higher contamination observed in the connectors as seen with other treatments. These results highlight the importance of thorough investigation of efficacy of disinfection methods and that endoscope design/geometry is a key consideration. Ultimately, the use of bacterial bioluminescence offers a valuable experimental model for understanding biofilm dynamics within the context of endoscopes and can aid improvement of reprocessing protocols.

## Data Availability

The original contributions presented in the study are included in the article/supplementary material, and further inquiries can be directed to the corresponding author.

## SUPPLEMENTAL MATERIAL

Supplemental Material aac.01380-25-s0001.docx Fig. S1 to S4.

ASM does not own the copyrights to Supplemental Material that may be linked to, or accessed through, an article. The authors have granted ASM a non-exclusive, world-wide license to publish the Supplemental Material files. Please contact the corresponding author directly for reuse.
