# Supplementary material for: Bacterial bioluminescence for the real time and in situ monitoring of endoscope disinfection using plasma activated water
Source: Antimicrob Agents Chemother. 2026 Jan 30;70(3):e01380-25. doi: 10.1128/aac.01380-25 (PMC12959089; doi:10.1128/aac.01380-25)
Supplement: Supplemental Material — Fig. S1 to S4. [file aac.01380-25-s0001.docx]

**Bacterial bioluminescence for the real time and *in situ* monitoring of endoscope disinfection using plasma activated water**

**Naomi Northage^1^, Joshua A. C. Steven^2^, Darren M. Reynolds^2^, Malcolm J. Horsburgh^3^, James L. Walsh^4^, Robin M. S. Thorn^2,*^**

^1^Department for Gaseous Electronics, Jožef Stefan Institute, Ljubljana SI-1000, Slovenia

^2^School of Applied Science, College of Health, Science and Society, University of the West of England, Bristol, BS16 1QY, UK

^3^Infection Biology & Microbiomes, Institute of Infection, Veterinary and Ecological Sciences, University of Liverpool, Liverpool, L69 7BE, UK

^4^York Plasma Institute, School of Physics, Engineering & technology, University of York, York, YO10 5DQ, UK


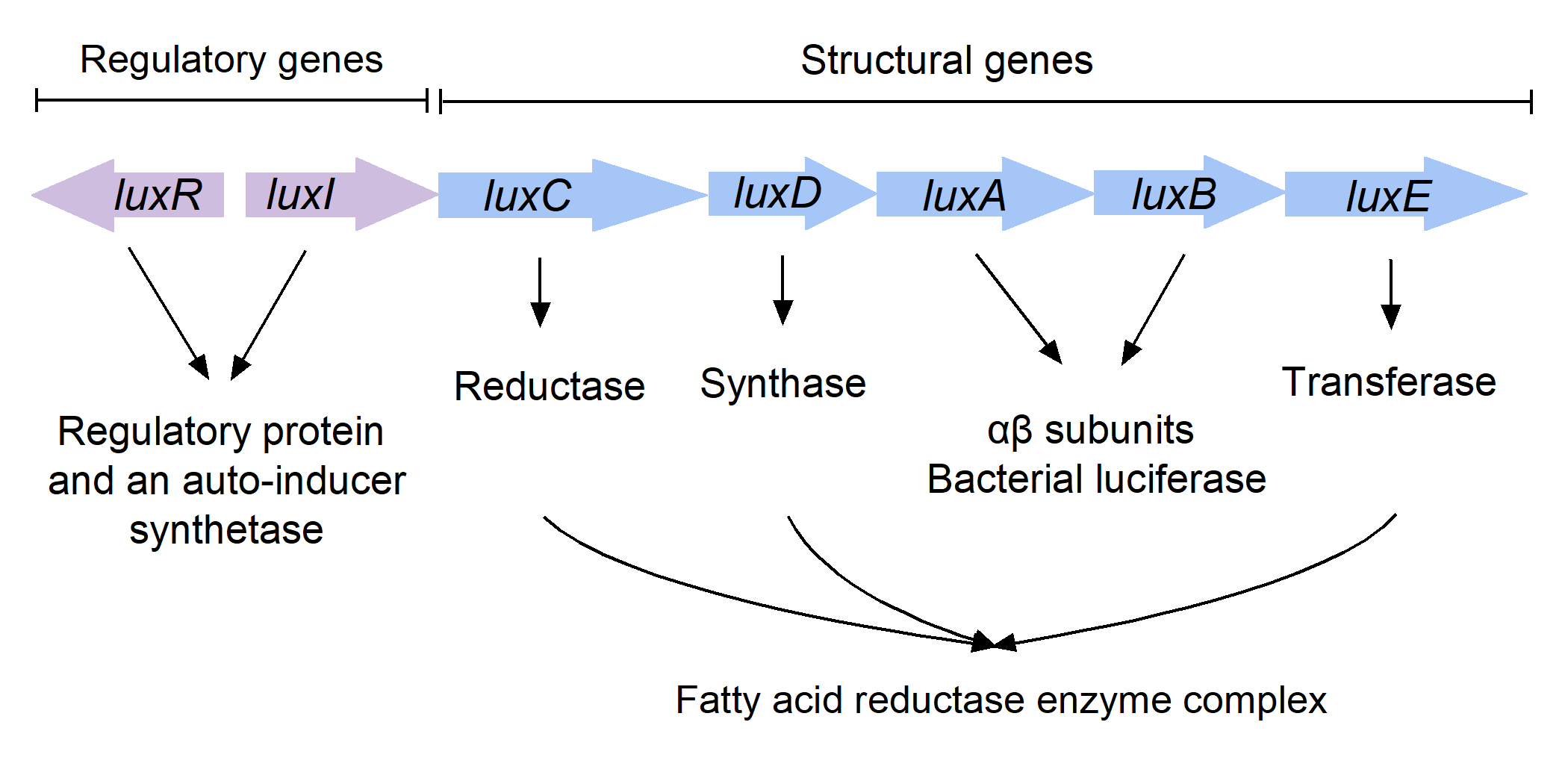


***Supplementary Figure S1.*** *The lux operon. The operon consists of two regulatory genes upstream of the luxCDABE gene cassette: luxR encoding a regulatory protein and luxI encoding an auto-inducer synthetase. The luxCDABE gene cassette consists of luxAB which encode alpha and beta subunits of a bacterial luciferase and the luxCDE genes encoding a fatty acid reductase enzyme complex****.*** *In the reporter construct, a constitutive reporter is used instead of luxR and luxI.*


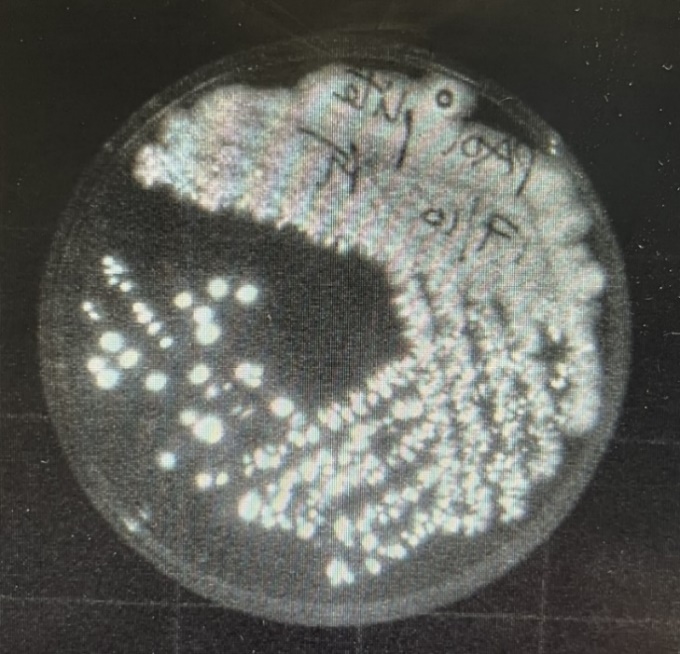


***Supplementary Figure S2.*** *Bioluminescence produced by the bacterial strain P. aeruginosa PAO1 SEI MCS5-lite (PAO1 plite), visualized using an EMCCD photon-counting camera in a dark room. The image of the agar plate confirms bioluminescence of the strain prior to experimental assays.*


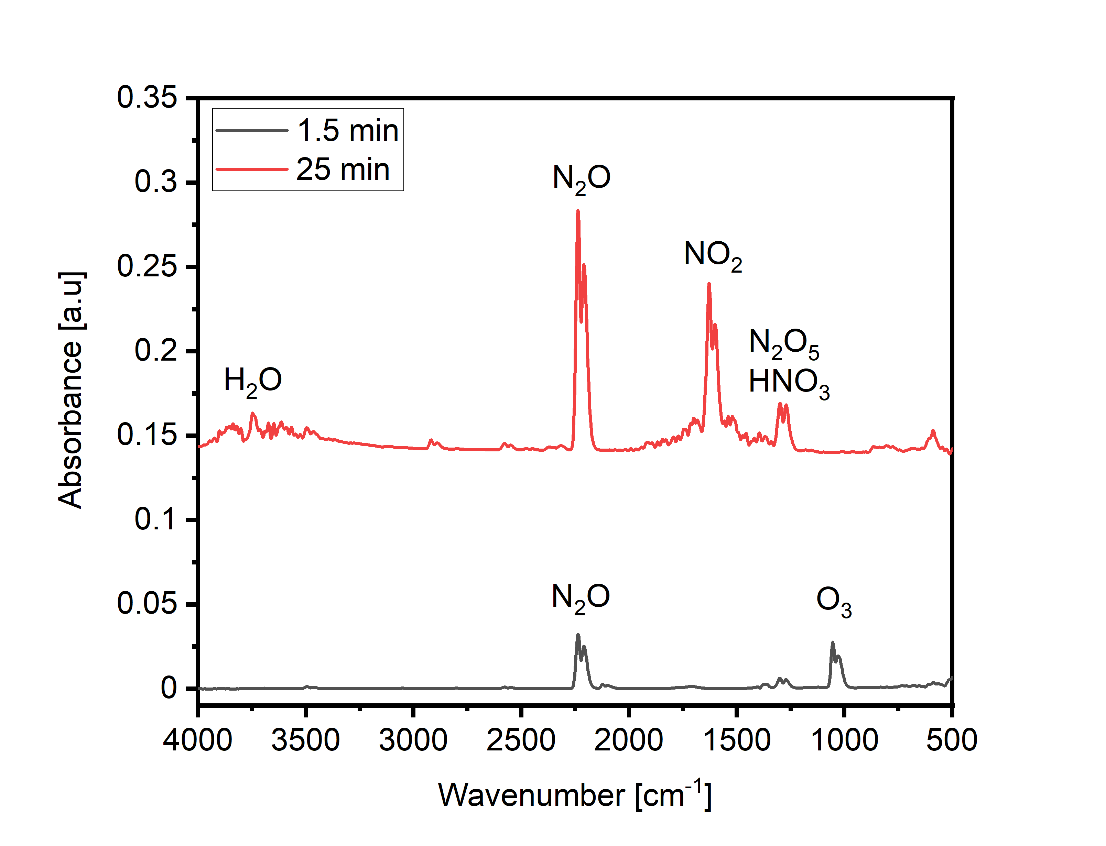


***Supplementary Figure S3.*** *Fourier-transform infrared (FTIR) absorbance spectra obtained after 1.5 and 25 minutes of plasma generation, demonstrating the chemical composition of the plasma effluent. The spectra were recorded with 25 scans for each measurement and at a resolution of 2.0 cm⁻^1^. The highlighted peaks correspond to groups of interest.*


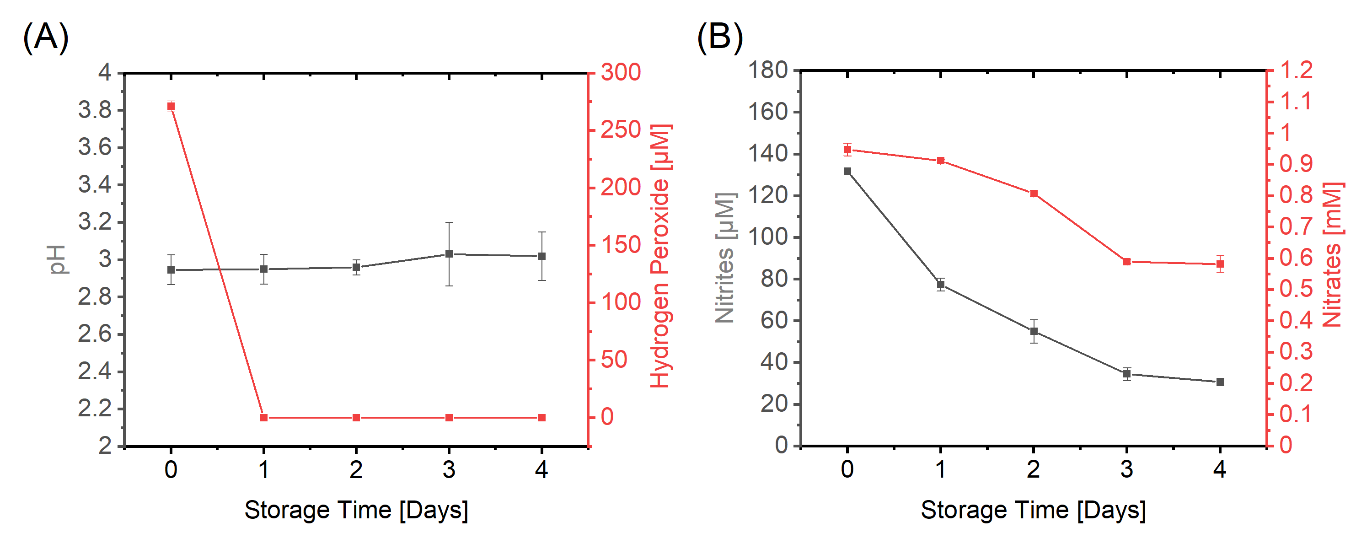


***Supplementary Figure S4.*** *Evolution of key components of the plasma activated water over a 4-day storage period. (A) Changes in pH and hydrogen peroxide (H₂O₂) concentration. (B) Concentrations of nitrites (NO₂⁻) and nitrates (NO₃⁻).*
